# Pesticide reduction amidst food and feed security concerns in Europe

**DOI:** 10.1038/s43016-023-00834-6

**Published:** 2023-09-21

**Authors:** Kevin Schneider, Jesus Barreiro-Hurle, Emilio Rodriguez-Cerezo

**Affiliations:** Joint Research Centre, European Commission, Seville, Spain

**Keywords:** Agriculture, Environmental studies

## Abstract

Recent studies have estimated the potential yield impacts of pesticide reductions in the European Union. While these estimates guide policy design, they are often based on worst-case assumptions and rarely account for positive ecological feedbacks that would contribute to sustainable crop yields in the long term.

## Abstract (web summary)

The European Commission’s recent proposal for more sustainable use of plant protection products has raised concerns over the impact that reduced pesticide use could have on agricultural yields. This Perspective reviews some of the impact assessments informing policy developments and critically analyses their assumptions.

## Main

The European Green Deal aims to reduce the use and risk of chemical pesticides, as well as the use of more hazardous pesticides, by 50% by 2030. The European Commission approved the proposal of the Sustainable Use of Plant Protection Products Regulation (SUR) in June 2022 to establish binding legislation on this Green Deal target. In December 2022, the UN Convention on Biological Diversity in the Kunming–Montreal Global Biodiversity Framework committed to reducing the overall risk from pesticides and highly hazardous chemicals by at least half, echoing the need for action.

The SUR has several objectives. First, reducing the use and risk of chemical pesticides generally, and the use of more hazardous substances. The two indices used to track these targets are aggregates that take pesticide sales and a weighting factor into account. The weighting depends on the risk class of each given active substance. The indices may need improvements^1^; nevertheless, the risk-based weighting has implications for national plans to reduce the indices (for example, via substitutions of active substances) that were not well captured in the analyses discussed below. Second, the SUR aims to increase the adoption of integrated pest management (IPM). Third, the SUR improves the availability of data on pesticide use and IPM implementation.

The SUR is one of the actions supporting the accelerated transition to a sustainable food system intended under the European Union (EU)’s Farm to Fork Strategy. The regulation contributes to the United Nation’s Sustainable Development Goals (SDG2, food security; SDG3, good health; SDG6, clean water; SDG8, economic growth; SDG12, responsible consumption and production; and SDG15, protection of life on land) and to various key policy strategies within the European Green Deal, such as the Biodiversity Strategy, the Zero-Pollution Action Plan, the Nature-Restoration Targets, the Soil Strategy, the Pollinators Initiative, the Groundwater and Drinking Water Directives and the Chemicals Strategy.

The SUR is an important policy milestone because it would be the first binding regulation to include pesticide reduction targets. While legislation on pesticide use is broadly supported by scientists^2^, in December 2022 the Council of the EU requested additional scientific evidence on the potential impacts of the SUR. The war in Ukraine and the resulting concerns about food and feed security for Europe were put forward as reasons why impacts had to be revisited. Despite the European Commission responding to the Council Decision^3^, voting on the SUR has been pushed back from July to October 2023. Here we comment on the potential implications of a pesticide reduction on crop yields as an important element of food and feed security in Europe to inform the political debate on the regulation.

**Figure:**
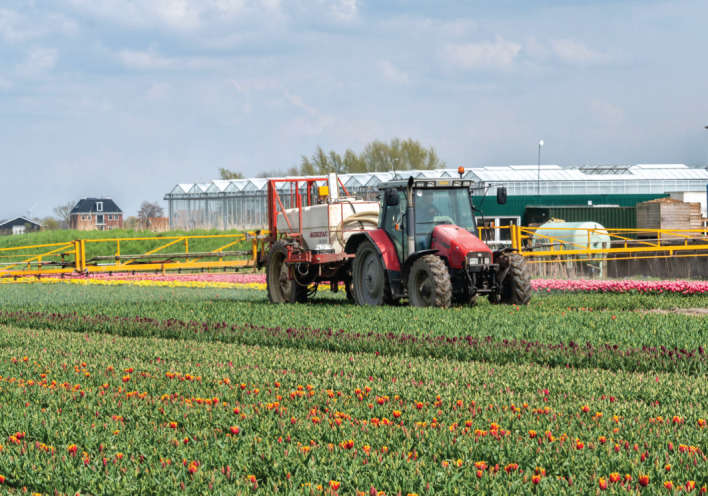
Steppeland / Alamy Stock Photo

## Studies suggest repercussions from a pesticide reduction

Several studies have analysed the potential impacts of the intended pesticide reduction on crop yields^4–9^. All have assumed that the SUR would result in a full 50% reduction in pesticide use and risk in all crops and all EU Member States. Owing to the lack of field data on yield impacts, these analyses either elicited estimates or imposed assumptions of adverse impacts on yields to inform economic simulations. Possible market changes were generally not included, such as changes in the products consumed^10^ and market-based policy instruments geared towards low pesticide use^11^.

Pesticide reductions were generally assumed to reduce crop yields, leading to higher food prices, increased imports and reduced exports of commodities. EU-wide impacts ranged from declines in production of 7.9% for cereals, 11.0% for oilseeds and 10.4% for vegetables in Barreiro-Hurle et al.^5^ to reductions in production of 21.4% and 20% for cereals and oilseeds, respectively, in Henning et al.^7^. Country-specific expert elicitations suggested yield reductions of 5% (France) to 10% (Germany) for wheat, 10% (Germany) to 13% (Poland) for rapeseed, 3% (France) to 15% (Germany) for sugar beets, 0% (France) to 4% (Romania) for maize, 8% (Italy) to 20% (Poland) for apples, 20% (Italy and Spain) for tomatoes and 13% (Spain) to 30% (Italy) for olives^6^. The yields of various vegetables under greenhouse cultivation may be reduced by up to 20% (ref. ^9^). If countries across the globe were to adopt various Green Deal targets, estimates suggest a 12% reduction in agricultural yields^4^.

Yield impacts would probably have different economic consequences for food and feed uses^3^. Commodity inputs generally account for a smaller share of food production costs. Here, processing, packaging and transportation are major cost drivers. Conversely, feed costs are generally a considerable share of the production costs for livestock. Price increases for crops due to lower yields would thus probably affect production costs for livestock more directly than those for crop-based food products.

While these analyses provided important insights under assumptions that reflect a possible implementation of the SUR, some of these assumptions indicate a simplistic understanding of how pesticide reduction targets could be implemented. Furthermore, the potential for alternative approaches to pesticides and positive ecological feedbacks on yields following lower pesticide use were not captured in these estimates.

## Pesticide reduction targets are more multifaceted

Reductions in pesticide use can be achieved in non-agricultural areas such as urban areas, private gardens, roads and railways, non-agricultural parts of ecologically sensitive areas, agricultural land devoted to non-productive features and so on. The SUR refers to these locations as ‘sensitive areas’ and specifically proposes a pesticide reduction for such uses. Reductions here will arguably have few implications for food and feed security in terms of reaching the target. While the size of this contribution probably varies across countries, the magnitude of pesticide use in non-agricultural areas is generally small compared with agricultural use. For a discussion on the sensitive areas and land-use data that depicts national differences, see chapter 4 in the Commission Response to the Council Decision (EU) 2022/2572 (ref. ^3^).

Reducing pesticide use and risk in non-food and non-feed sectors is critical. In developed economies, the aggregate of the pesticide footprint linked to the consumption of textiles, services, other and ‘empty-calorie food’ (that is, foods that have little to no nutritional value) accounts for a share of 37% (ref. ^10^). This estimate does not account for pesticide use in non-cropland (for example, urban areas). Countries could therefore prioritize other sectors in their national reduction plans, again limiting potential repercussions on food and feed security by reducing the share of the 50% reduction target that falls onto food and feed crops.

A flat-rate pesticide use and risk reduction of 50% across all crops and areas seems unlikely. The pesticide intensity varies considerably across crops and regions. This allows countries to target specific crops that contribute more strongly to their current aggregate pesticide use and risk, or target crops for which management alternatives are available. As different countries may prioritize different crops, even under the assumption of adverse effects on yields, the crop-specific supply shocks across the EU would be considerably more spread over commodities than is captured by existing studies on impacts. This would reduce the effects on production, prices and trade compared with what was simulated. The farm-level pesticide logbooks proposed in the SUR will enable crucial work on country-specific prioritizations of crops, areas and sectors.

## Heterogeneity between farmers implies a potential for efficiency gains

Pesticides use intensity depends on many aspects, ranging from (1) biological factors such as pest abundance, local climate, soil type and regional crop diversity to (2) agronomic factors such as decisions on tillage, sowing date, variety susceptibility, fertilization and crop rotation^12^, (3) economic factors such as the expected yield and the on-farm economic and financial situation^13^ and (4) social, as well as political, factors^14^. The multitude of relevant factors results in considerable spatial heterogeneity in pesticide use on a global scale^15^ and even in geographically small countries^16^. Globally, around one-third of the cross-country differences in pesticide pollution risk are linked to differences in the underlying food systems and pesticide regulations^17^. Various studies have found sizeable variability in pesticide use not only across different years and regions within a country, but also across farms that faced the same environmental and socio-economic conditions^12,13,18,19^. Potential gains in efficiency were not accounted for in the impact analyses.

Under the SUR, the farm-level pesticide logbooks will address critical data gaps^20^. These data will, for example, enable the benchmarking of pesticide use at the farm level to identify heterogeneity in pesticide use, which could inform interventions. Pesticide data may also improve decision support systems, which can considerably lower pesticide needs without increasing disease risk and without yield reductions^21,22^. Technological advances enable the targeted applications of pesticides^23^, considerably improving the efficiency^24^.

## Input substitution enables progress towards pesticide reduction targets

As the indices are risk-based, the substitution of active substances enables progress towards the target, and may deliver environmental and social benefits without affecting the availability of chemical management tools for farmers. By 2021, a reduction of 33% and 21% in the two targets from the reference period (2015–2017) was achieved at EU level, mostly due to substance substitution (https://food.ec.europa.eu/plants/pesticides/sustainable-use-pesticides/farm-fork-targets-progress/eu-trends_en). Substitutions of active substances may be made by other chemical pesticides with a lower toxicity^24^, via biopesticides^25^ or by using alternative approaches such as biological control with antagonists, predators or parasites of plant pests^26^.

IPM is ideally based on varieties with biotic resistances. Varieties with ‘stacked’ resistances enable a system-wide rethinking of crop protection, which allows significant reductions of pesticides^27^. Breeding for biotic resistance may be encouraged by the need to implement the SUR targets. New breeding technologies may contribute to this growth by the availability of resistant varieties. The European Commission’s proposal on new legislation for ‘New Genomic Techniques’ may facilitate access to improved varieties that could substitute existing ones in the near future^28^.

## Redesign of managerial approaches supports a systemic transformation

A successful transition towards lower pesticide use in agriculture must build on the diversity of knowledge on complementary strategies for crop protection as formulated in the IPM principles^29^. The availability of such practices is ample with over 1,300 IPM strategies and 270 crop-specific guidelines from 24 EU countries recently compiled into a common database (https://datam.jrc.ec.europa.eu/datam/mashup/IPM/index.html) within an EU-funded project entitled Farmer’s toolbox for IPM. While IPM is already mandatory in the EU, the SUR aims to increase the availability of crop-specific guidelines and the record-keeping of IPM to improve enforcement and monitoring. Unintended consequences, such as soil erosion and fuel consumption under mechanical weeding, must be considered when redesigning managerial processes^30^.

A sound integration of agro-ecological principles, both at the field and landscape levels, supports the prevention of pest and disease impacts^31,32^. At the farm level, various agronomic decisions may determine the resilience to pests, and in turn the need to apply pesticides. Farmers generally practice a temporal rotation of crops that allows optimization of nutrient use, a reduction of pests and improvements to the soil biota with feedbacks to crop yields^33^. Similarly, spatial configurations (for example, intercropping, strip-cropping) of hosts can support natural pest control at the field and landscape levels^32,34–36^. Complex landscapes, in turn, can lower pesticide needs^37^ and support the substitution of pesticides with biological pest control^38^. Potential negative economic impacts due to limiting the economies of scale must be carefully assessed.

Synergistic targets must be considered in political discussions of individual regulations such as the SUR. Organic production rules, including the use of pesticides, are laid down in EU legislation (Regulation (EU) 2018/848) (ref. ^39^). In Europe, organic farming is generally associated with a reduction in the use of chemical pesticides^17,40^. The Farm to Fork strategy set a clear target of increasing the area under organic farming from the current level of 12% to 25% by 2030. A relevant effect of this expansion will be a reduction in the use of agricultural inputs such as chemical pesticides. Converting 25% of the area to organic farming may decrease pesticide purchases by 14.5%, which could deliver 5% of the pesticide target^8^. However, this contribution remains uncertain due to the target being non-binding, and may come at an expense of production due to the lower yields associated with organic farming^41^. The transition to more area under organic farming does warrant a careful analysis on food security impacts that may arise.

## Reduced pesticide use contributes to sustainable yields in the long term

Besides the intended function of pesticides as damage control agents, their use also leads to unintended consequences, so-called externalities^42^. The unprecedented loss of biodiversity threatens food systems globally, putting food and feed security severely at risk^43^. By supplying many vital ecosystem services, a rich biodiversity makes food systems more resilient to shocks and stressors^44^, including those caused by climate change. The current use of pesticides has been established to be a key driver of biodiversity loss across the EU^42^. The adverse effects of pesticides on biodiversity are not limited to lethal doses, but also arise through a continuous exposure at sub-lethal levels^45^. Consequently, pesticide use generates negative externalities that are relevant for food and feed security concerns; for example, a reduction in natural pest control, pollination services and soil health^46^.

Pollinators influence 35% of the global human food supply^43,47^. Despite this, an EU-wide ecosystem assessment revealed that 50% of the land cultivated with pollinator-dependent crops faced a deficit in pollinators. Many soil ecosystem services are biologically mediated^42^. In turn, the effects of pesticides on the soil biota affect, for example, the efficiency of nutrient cycling and productivity, which in turn negatively influence yields and resilience to extreme weather events. This can lead to increased impacts of climate events and higher volatility of crop yields^48^, aggravating climate change-related risks to food and feed security. A reduction of pesticide use and risk would therefore contribute to sustainable crop yields in the long run. The time needed to observe tangible benefits from improved ecosystem services probably varies by environmental compartment and organism, but certainly requires a long-term societal commitment.

## Improved scientific approaches can better inform political decision-making

A successful pesticide policy framework requires, among other considerations, reflections on the indices, approaches to active substance approval and the decision-making processes at the farm and consumer levels^49^. For actors along the food chain to engage fully, evidence that addresses distinct motivations and concerns across the diversity of stakeholders will be needed^50^.

Evidence-based decision-making is challenged by current limitations in scopes, conceptual designs and methodologies^51^. The multifaceted nature of the Green Deal target requires a rethinking of sound modelling approaches that capture not only potential economic outcomes, but also the environmental and social dimensions of the policy objectives, as well as their interlinkages. The missing feedbacks between improved functional biodiversity and crop yields, following lower pesticide use and risk, in the impact studies discussed here is a clear example of the repercussions modelling limitations can have for the political debate. In addition to much-needed methodological advances, reimagining data collection, for example through on-farm experimentations^52^, could enable more integrated research and simultaneously foster the engagement of stakeholders.

Protecting crop yields is critical to safeguarding food and feed security. Studies on the potential yield impacts of a reduction in pesticide use and risk in the EU estimated adverse effects. As shown here, the literature suggests that these estimates are upper bounds for several reasons that must be acknowledged in research on the impacts of a pesticide reduction: the full 50% reduction does not fall onto feed and food crops; the heterogeneity in pesticide use across farms, areas and crops can be exploited in reduction plans; risk-based indices allow for progress by substituting active substances; the expansion of the area under organic farming may deliver progress; the SUR facilitates agronomic and technological alternatives to pesticides; and ecosystem services supporting sustainable crop yields will benefit from lower pesticide use. Finally, the SUR improves the availability of data on pesticide use and, in doing so, addresses a bottleneck in research and policy-making concerning more sustainable food systems.
